# Vimentin Overexpressions Induced by Cell Hypoxia Promote Vasculogenic Mimicry by Renal Cell Carcinoma Cells

**DOI:** 10.1155/2019/7259691

**Published:** 2019-07-21

**Authors:** Hao Lin, Yingkai Hong, Bin Huang, Xincheng Liu, Junhong Zheng, Shaopeng Qiu

**Affiliations:** ^1^Department of Urology, The Second Affiliated Hospital of Shantou University Medical College, China; ^2^Department of Urology, The First Affiliated Hospital of Shantou University Medical College, China; ^3^Department of Urology, The First Affiliated Hospital, Sun Yat-sen University, China

## Abstract

Vasculogenic mimicry (VM), the novel approach for tumor cells to obtain blood supply, was reported to be involved in antiangiogenic resistance and poor prognosis in renal cell carcinoma (RCC). However, the molecular mechanisms underlying VM formed by RCC cells are still not clearly depicted. In the present study, we found that OS-RC-2 acquired the VM forming ability accompanied with the increased expressions of Vimentin and AXL and decreased expression of E-Cadherin by CoCl_2_ treatment. Downregulation of Vimentin by siRNA severely impaired the capability of OS-RC-2 and 786-O to form VM structures induced by cell hypoxia in vitro. Moreover, knockdown of Vimentin inhibited cell migration and invasion, which could be prompted by hypoxia induction in RCC cells. In our clear cell RCC tissues, we found that VM was positively correlated with Vimentin overexpression and both predicted poor prognosis. In conclusion, Vimentin plays an important role in hypoxia induced VM formation of RCC cells and targeted Vimentin might be beneficial for RCC therapy.

## 1. Introduction

Renal cell carcinoma (RCC) is among the most common cancers around the world [1]. It accounts for 4% of all adult malignancies in the USA in 2017 [2]. While 65% of patients with localized disease can be treated with surgery by total or partial nephrectomy, the rest of 35% who presented with metastatic RCC (mRCC) or those who relapsed after local therapy require systemic therapy [3, 4]. Although the management of mRCC has changed dramatically as a result of developments in target tumor vasculature therapy over the past few years, a large subset of patients treated with antiangiogenic agents will eventually experience drug resistance and disease progression [1]. Heterogeneity in RCC changes over time in response to therapy might partially explain acquired resistance [5, 6]; more and more clinical and preclinical evidence shows that resistance is mediated by tumor cells and tumor microenvironment [7–10]. But the exact underlying mechanism is yet to be determined.

Vasculogenic mimicry (VM), the mechanism by which tumor cells acquire endothelial cell phenotype and contribute to metastasis, is reported to be involved in antiangiogenic resistance [11, 12]. Recently, Maria Serova et al. found that VM was associated with sunitinib resistance and a more aggressive phenotype in* in vitro* and* in vivo* RCC models; moreover, they observed increased expression of Vimentin during sunitinib treatment in a xenograft model [13]. Vimentin is a major constituent of the intermediate filament (IF) family of proteins and also a marker of epithelial-mesenchymal transition (EMT) (reviewed in [14]). Although EMT has been demonstrated to participate in VM in a variety of cancers (reviewed in [15]), the role of Vimentin underling this mediating process in RCC remains unknown.

In RCC, Vimentin overexpression is one of the independent predictors of poor clinical outcome and may serve as a useful adjunct in differentiating different pathology types [16, 17]. By virtue of its unique expression pattern in RCC, Vimentin may serve as an attractive target for RCC therapy. Further study directed toward elucidating the role of Vimentin in RCC cells VM might open up new approaches for developing promising therapeutic drugs.

In the present study, we concentrated on defining the specific role of Vimentin induced by cell hypoxia in VM formed by RCC cells. We showed that cell hypoxia may contribute to VM forming ability of RCC cells through EMT, characterized by enhancement of Vimentin and AXL and decrease of E-Cadherin expressions. In addition, we showed that downregulation of Vimentin expression reduced RCC cell invasion and migration and VM formation. Finally, we validated the correlation of VM and Vimentin expression in RCC tissues and their association with clinical parameters.

## 2. Materials and Methods

### 2.1. Ethics Statement

This study was approved by the Medical Ethics Committee of Sun Yat-sen University, and written informed consent was obtained from each patient for surgery and research purposes.

### 2.2. Cell Culture and Hypoxia Mimicking

The RCC cell lines 786-O, 769-P were obtained from American Type Culture Collection and kept in RPMI-1640 (Gibco, USA) supplemented with 10% fetal bovine serum (FBS) (Gibco, USA). The OS-RC-2 was a kind gift from Dr. Xu Chen (Department of Urology, The First Affiliated Hospital, Sun Yat-sen University) and was also maintained in RPMI-1640 (Gibco, USA) supplemented with 10% fetal bovine serum (FBS) (Gibco, USA). All the cells were kept in a 37°C humidified incubator with 5% CO_2_. The day before hypoxia induction, the media of cells were changed to RPMI-1640 without serum and cultured for 24h. The 786-O and OS-RC-2 were incubated for different time periods (24h to 72h) in the absence/presence of cobalt chloride (CoCl_2_, final concentration=200*μ*M) (Sigma, USA) which is a widely used hypoxia mimicking agent [18].

### 2.3. VM Assay* In Vitro*

Ninety-six well plates were coated with growth factor reduced Matrigel (BD Biosciences, Bedford, MA, USA) (60*μ*l/well), which was allowed to polymerize for 1h at 37°C. Then cells at a density of 4×10^5^/ml were seeded on wells coated with solid Matrigel with/without CoCl_2_. The observation time for 786-O and 769-P was 4h, and for OS-RC-2 it was between 24 and 72h. Photographs were taken and the numbers of complete tubular structures from three randomly chosen fields were counted. The mean value of the three readings was used as the final reading of the well.

### 2.4. RNA Extraction and Quantitative-PCR

Briefly, the total cellular RNA was isolated using PureLinkr® RNA Mini Kit (Ambion, USA), reversely transcribed to cDNA using RevertAid First Strand cDNA Synthesis Kit (Thermo Scientific, USA), and Quantitative-PCR (TaKaRa Biotechnology Co., Ltd., Japan) analysis according to the manufacturers' instructions. The forward primer for Vimentin was 5′-CCGACACTCCTACAAGATTTAGA-3′, and the reverse primer was 5′-CAAAGATTTATTGAAGGAGAACC-3′. The forward primer for *β*-actin was 5′- AGCGAGCATCCCCCAAAGTT-3′, and the reverse primer was 5′-CAAAGATTTATTGAAGGAGAACC-3′.

### 2.5. Western Blot

Briefly, the whole cell lysates were separated by 8% sodium dodecyl sulfate-polyacrylamide gel electrophoresis and transferred onto polyvinylidene difluoride membranes (Millipore, German). Blots were blocked with 5% fat-free milk overnight at 4°C and incubated with antibodies for 1-3h at room temperature. Then blots were incubated with a horseradish peroxidase-conjugated goat anti-rabbit secondary antibody (1:5000, Abcam, USA) and imaged by chemiluminescence (ECL) (Bio-Rad, USA) working solution. The details of antibodies using here were as follows: Vimentin (1:1000, CST, USA); AXL (1:1000, Abcam, USA); E-cadherin (1:1000, CST, USA); *β*-actin (1:5000, Abbkine, USA).

### 2.6. Cell Transfection

Cells were transfected with siRNA targeted Vimentin (sc-29522, Santa Cruz, USA) using lipo2000 and Opti-MEM I (Gibco, USA). Six hours after transfection, siRNA were removed by changing the medium with RPMI-1640 with 10% FBS with/without CoCl_2_. Cells were cultured for additional 48-72 h and then harvested for Western blot testing.

### 2.7. Wound Healing and Invasion Assays

For wound-healing assay, cells were cultured in 6-well plates. Twenty-four hours before the experiment, cells were cultured with serum-free RPMI-1640 with/without CoCl_2_. When the cells grew to confluence, a straight scratch was made in the center of each well using a micropipette tip, and the cells were washed with PBS and incubated in serum-free medium with/without CoCl_2_. The initial gap length and the residual gap length at 0 h, 24 h after wounding were observed under an inverted microscope and captured. The wound area was measured by the program Image J (http://rsb.info.nih.gov/ij/). The percentage of wound closure was estimated by 1 - (wound area at Tt/wound area at T0) × 100%, where Tt is the time after wounding and T0 is the time immediately after wounding.

For invasion assay, 500*μ*L of prepared serum-free suspension of cells (1 × 10^5^ cells/mL) with/without CoCl_2_ was added into the upper insert (8*μ*m pore size, Corning, USA); 500*μ*L of medium containing 10% fetal bovine serum was added to the lower chamber of the insert. Cells were allowed to invade for 48h. Then, noninvading cells in the upper insert were gently removed with a cotton tipped swab; invasive cells on the lower surface of the inserts were stained with the staining solution for 20min and counted under a microscope. Each experiment was performed in triplicate.

### 2.8. Patients and Tissues Samples

A total of 113 patients with ccRCC from the First Affiliated Hospital of Sun Yat-sen University between 2010 and 2012 were enrolled in this study. All these patients did not receive any treatments before radical nephrectomy or partial nephrectomy. ccRCC was confirmed by pathology findings. Patients were followed up by clinic interview or telephone. The total followed up period was from 9 to 60 months. Overall survival (OS) time was calculated as the duration from the date of surgery to the date of death. Of the 113 samples, 77 were male and 36 were female, and the median age was 55 years (range: 30 years to 80 years). Tumors were staged according to the 2009 TNM staging system [19] and graded according to the criteria of the World Health Organization [20, 21].

### 2.9. Immunohistochemistry (IHC)

Sections of 4*μ*m in thickness were mounted on slides. After dewaxing, rehydration, and antigen retrieval, the slides were then incubated with polyclonal rabbit IgG anti-Vimentin antibody (1:100, CST, USA). All sections were accessed and scored by two independent pathologists according to staining intensity and the percentage of positive tumor cells as previously described. High expression of Vimentin was defined as staining index ≥4 while low expression of Vimentin was defined as staining index <4.

### 2.10. CD34/PAS Double Staining

CD34 IHC was applied to the slides prior to PAS staining. Monoclonal CD34 antibody (1:100, Abcam, USA) was used in the IHC staining. The sections were rinsed with distilled water and then treated with 0.5% periodic acid solution for 10min and rinsed with distilled water again for 5min. The slides were kept in a dark chamber and treated with Schiff solution for 15–30 min. After distilled water rinsing, slides were counterstained with hematoxylin. The results were quantified as our previous study [22].

### 2.11. Statistical Analysis

Statistical data were evaluated using SPSS21.0 (IBM Corp. Armonk, NY, USA) and Graphpad prim5 (Graphpad Software, Inc., USA). All experiments were repeated for at least three times. Continuous data were expressed as the mean ± standard deviation (S.D.) and analyzed by t-test or one-way analysis of variance (ANOVA). The relationship between VM and Vimentin expressions and clinical parameters were accessed by *χ*^2^ or Fisher's exact test. The correlation between VM and Vimentin was accessed by spearman test. Survival analysis was estimated by Kaplan-Meier method and differences in survival were tested by log-rank test. Statistical significance was set at* P*<0.05.

## 3. Results

### 3.1. Cell Hypoxia Promoted VM Formation* In Vitro* in OS-RC-2

**Figure 1:**
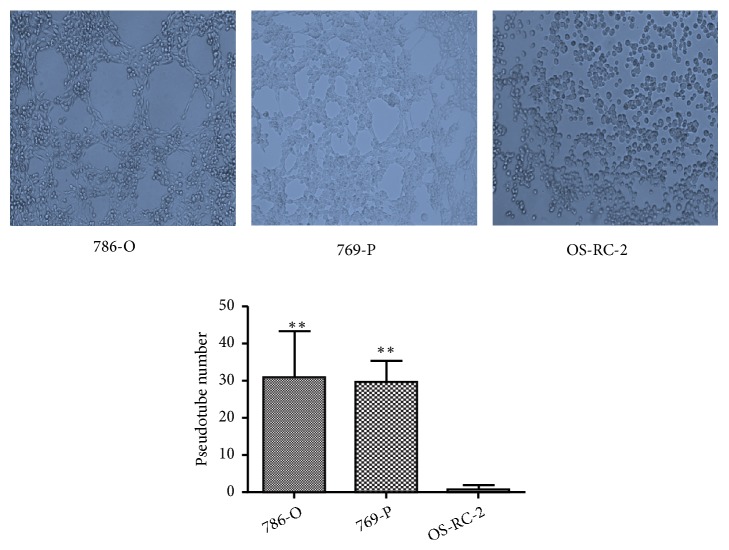
VM forming ability under normoxia of RCC cell lines. Under normoxia condition, 786-O (a) and 769-P (b) were able to form tubular structures on Matrigel when seeded with a concentration of 4×10^5^ cells/ml for 4h. However, OS-RC-2 (c) did not show the VM forming ability in the normal circumstance; it was even seeded at a higher density and cultured for a longer period of time (×100).

**Figure 2:**
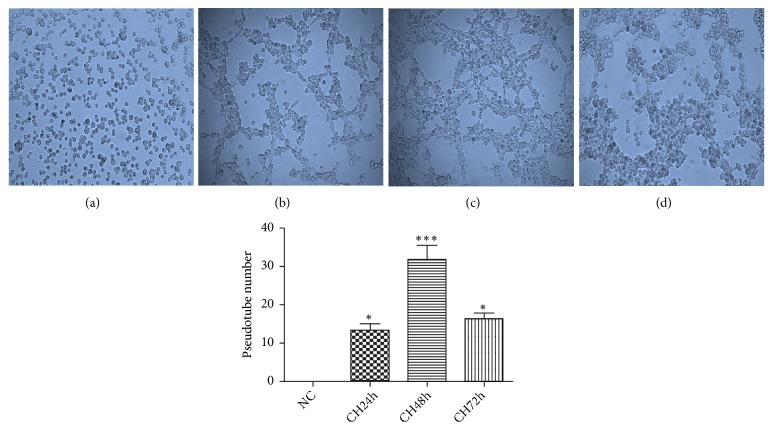
CoCl_2_ treatment on OS-RC-2. OS-RC-2 was cultured on Matrigel with CoCl_2_ (200*μ*M) for 0h (a), 24h (b), 48h (c), and 72h (d). By treating with CoCl_2_, numbers of pipe-like channels formed by OS-RC-2 began to increase at 24h and peaked at 48h. Interestingly, these VM structures started to disappear at 72h hypoxia induction (*∗*, P<0.05; *∗∗∗*, P<0.001) (×100).

In our previous study, we found that RCC cell lines 786-O and 769-P were able to form tubular structures on Matrigel* in vitro* in a cell number and cultured time dependent manner [22]. In comparing with these two cell lines, OS-RC-2 did not show the VM forming ability under normoxia condition even while being seeded with a higher cell concentration and cultured with a longer period of time up to 72h (Figure 1). We then examined whether hypoxia can stimulate VM formation of OS-RC-2. Cells cultured on Matrigel were incubated with CoCl_2_ for 24h, 48h, and 72h. Interestingly, OS-RC-2 began to form VM tubes 24h after hypoxia induction. By 48h culture under hypoxia condition, OS-RC-2 emerged with the maximum number of network patterns which began to reduce when hypoxia was prolonged to 72h (Figure 2).

### 3.2. Cell Hypoxia Increased Vimentin Expressions in OS-RC-2

**Figure 3:**
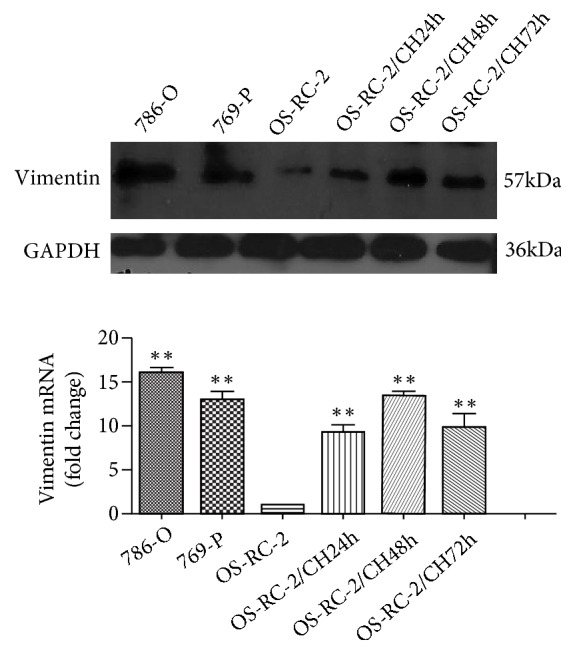
Vimentin expressions in RCC cell lines. Vimentin protein (a) and mRNA (b) expressions were higher in 786-O and 769-P than those in OS-RC-2 under normoxia. Noteworthy, cell hypoxia induced by CoCl_2_ treatment increased the expression of Vimentin on both mRNA and protein levels (*∗∗*, P<0.01).

To clarify the potential association of Vimentin with cell hypoxia, we first performed the RT-qPCR and Western Blotting to evaluate the expression of Vimentin in RCC cell lines in either normoxia or hypoxia. As shown in Figure 3, the expression levels of Vimentin were significantly higher in 786-O and 769-P in comparison with in OS-RC-2 under normoxia. By treating with CoCl_2_, the mRNA and protein levels of Vimentin in OS-RC-2 exhibited expression peaks 48h after cell hypoxia. These results implied that cell hypoxia may increase the expression of Vimentin in OS-RC-2.

### 3.3. VM Formation and Cell Invasion and Migration Were Impaired by Downregulation of Vimentin in RCC Cell Lines

**Figure 4:**
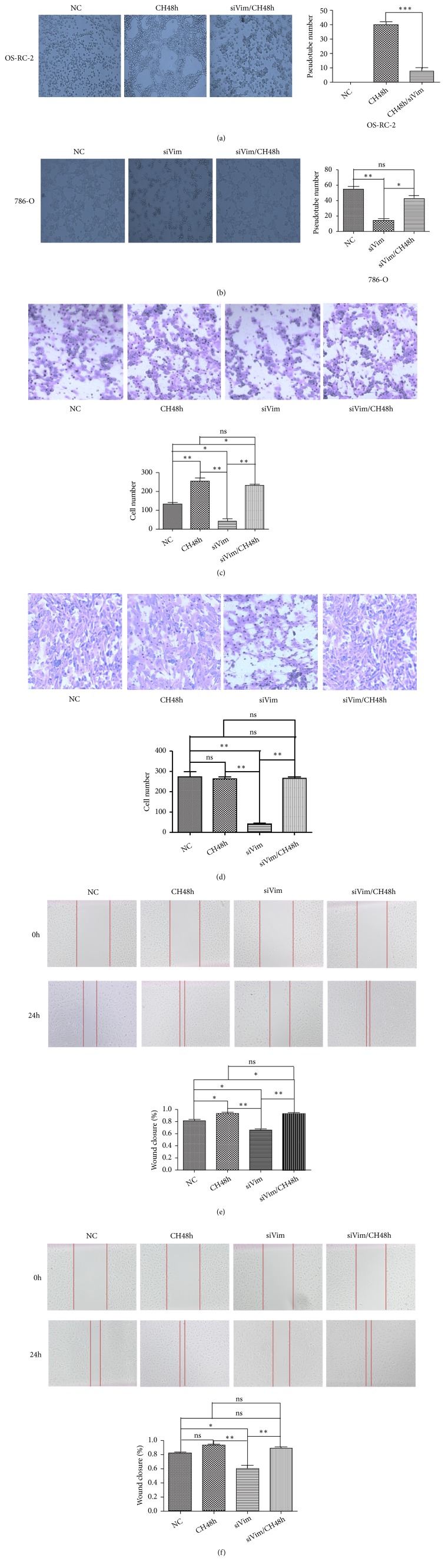
The effects of siVimentin and cell hypoxia on 786-O and OS-RC-2. While downregulation of Vimentin reduced cell migration and invasion and VM structures, cell hypoxia can reverse these effects of siVimentin ((a), (c), and (e) showing OS-RC-2; (b), (d), and (f) showing 786-O; CH48, cell hypoxia for 48h; ns, not significant; *∗*, P<0.05; *∗∗*, P<0.01; *∗∗∗*, P<0.001).

To further identify the role of Vimentin in VM, OS-RC-2 was treated with siRNA specifically to downregulate the expression of Vimentin with or without CoCl_2_. We observed that, while cell hypoxia induced the increases of VM and cell invasion and migration, siVimentin could weaken these effects of hypoxia in OS-RC-2 (Figures 4(a), 4(c), and 4(e)). In 786-O, reduction of VM and cell invasion and migration by knocking down the expressions of Vimentin could be overturned by induction of cell hypoxia (Figures 4(b), 4(d), and 4(f)). Taken together, these findings may imply that cell hypoxia promoted VM via the increase of Vimentin expressions.

### 3.4. Knocking Down of Vimentin Expressions Reversed Enhancement of AXL and Decrease of E-Cadherin Expressions Induced by Cell Hypoxia

**Figure 5:**
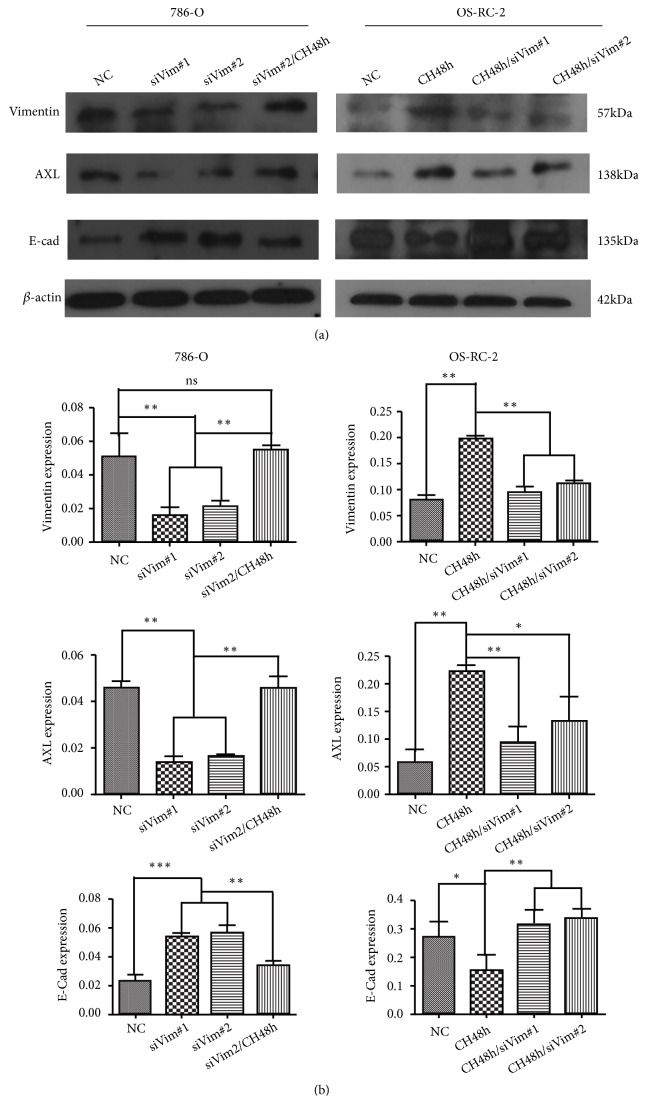
The effects of cell hypoxia on molecular signals in 786-O and OS-RC-2. By western blot, cell hypoxia increased the protein expressions of Vimentin and AXL and reduced the protein expression of E-Cadherin; knockdown of Vimentin was accompanied with suppression of AXL and increase of E-Cadherin expressions (CH48, cell hypoxia for 48h; ns, not significant; *∗*, P<0.05; *∗∗*, P<0.01; *∗∗∗*, P<0.001).

Hypoxia is sufficient to induce EMT* in vitro*, which is characterized by changes of cadherin protein expressions (reviewed in [23]). In the present study, immunoblotting of Vimentin, AXL and E-cadherin confirmed an EMT-associated shift as increase of Vimentin and AXL and decrease of E-cadherin expressions. Moreover, by knocking down the expression of Vimentin, we observed the reduced AXL was accompanied with the upraised E-cadherin (Figure 5).

### 3.5. VM Was Positively Correlated with Vimentin Overexpression and Both of Them Predicted Poor Overall Survival (OS) in RCC

**Figure 6:**
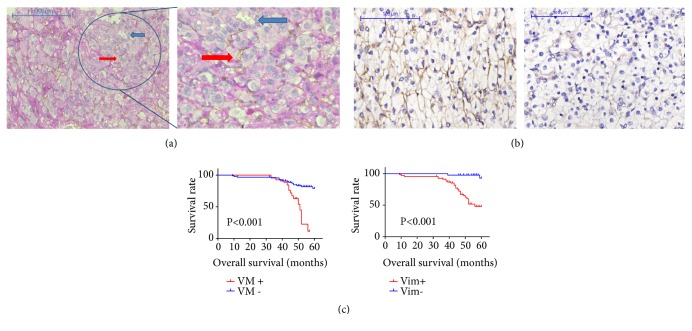
(a) VM phenomena in ccRCC tissues. The red arrows indicated microvessels with CD34 positive staining and the blue arrows indicated VM structures with CD34 negative and PAS positive staining. (b) Vimentin expressions in ccRCC tissues. Showing high expression of Vimentin (left) and low expression of Vimentin in ccRCC. (c) Both VM (left) and Vimentin expressions (right) were correlated with survival of ccRCC patients.

Previously, we showed that VM was mainly detected in ccRCC [22]. Similarly, Vimentin was reported to be expressed in nearly 90% of ccRCC [24] and could be a marker in distinguishing ccRCC from chromophobe RCC [25]. Here we tried to verify the relationship between VM and Vimentin in 113 ccRCC tissues by IHC (Table 1). VM was defined as PAS-positive and CD34 negative cavities with or without red blood cells in them. In the present study, VM was found in 26/113 (23.01%) of ccRCC samples (Figure 6(a)). VM was significantly associated with high TNM stage, tumor grade, and metastasis (all P<0.001). Vimentin was detected in 69 out of 113 ccRCC tissues and significant associations were observed between the positive expressions of Vimentin and clinical stage, pathological grade, and metastasis (all P<0.001) (Figure 6(b)). However, both of VM and high expression of Vimentin did not show positive association with sex, age, or location (all P>0.05).

The results also showed that all of the tumors with VM structures had a higher expression of Vimentin. Furthermore, we found that high Vimentin expressions were positively correlated with VM formation in our ccRCC samples (r=0.437, P<0.001). Kaplan–Meier survival analysis revealed that VM positive patients and those with Vimentin overexpression had shorter OS than those without VM and Vimentin overexpression (Figure 6(c)).

## 4. Discussion

Accumulated clinical investigations have shown that hypoxia was a characteristic feature of solid tumors that contributed to disease progression and therapy resistance [26]. Under anoxic environment, tumor cells may be adaptive to oxygen changes and develop into multipotent phenotypes [27]. Therefore, these plastic tumor cells may be engaged in VM formation, which was referred to the plasticity of aggressive tumor cells [28]. Indeed, more and more research shows that VM seemed to be related to hypoxia [29]. However, to our knowledge, none has explored the relationship between hypoxia and VM in ccRCC. Here, we first reported that cell hypoxia mimicked by CoCl_2_ treatment could induce OS-RC-2, which was unable to form VM under normoxia condition, to become VM formable cells on Matrigel* in vitro*.

Hypoxia is an inducer of epithelial mesenchymal transition (EMT), which is characterized by gain of migratory ability and loss of cell to cell junctions [30]. These changes triggered a cascade of cellular responses and finally may result in the formation of VM by invasive cancer cells through remodeling of external cellular matrix (ECM) [31]. Vimentin, a 57kDa protein of the type III IF family and a canonical marker of EMT [14], has been demonstrated to be involved in VM in a variety of cancers [18, 32, 33]. In line with these studies, we found that Vimentin expression was higher in the VM formable cells (786-O and 769-P). Furthermore, we observed an increase of Vimentin expressions in OS-RC-2 by hypoxia induction, which was accompanied with the acquisition of VM forming ability of OS-RC-2. This may suggest that Vimentin plays a pivotal role in VM formation of RCC cells.

Although Vimentin is critical in maintaining cell structures and may promote tumor progression, it is seemingly not an essential factor for survival under normal physical condition [34, 35]. In RCC, Vimentin was a specific marker in differentiating ccRCC from chRCC [36]. Taken together, Vimentin may serve as an excellent target for ccRCC therapy. In the present study, we found that, by knocking down the expression of Vimentin with siRNA, cell invasion and migration and VM structures were significantly impaired in RCC cell lines* in vitro*. Although there are a few reports showing the anticancer property of inhibition of Vimentin expression, specifically by downregulating cell migration and invasion [37, 38], none has indicated its effect on VM formation. Our findings may provide the rationale that, by utilizing vimentin as an anti-VM target, there will be a pertinent opportunity to overcome the current predicaments in RCC therapy.

We showed that cellular signaling responds to hypoxia through upregulation of Vimentin and AXL and downregulation of E-cadherin. Furthermore, we detected that the VM destruction by siVimentin was accompanied with reversed expression of AXL and E-cadherin. In fact, Vimentin has been shown to function as a regulator of AXL which enhanced cell migration [39]. We presumed that VM formation induced by cell hypoxia might be the effect of activation of Vimentin/AXL axis in RCC cells. However, further investigations are still needed in exploring the underlying mechanism.

ccRCC is the most common subtype of RCC [1]. Moreover, ccRCC accounts for the majority of deaths and is the predominance of metastatic disease in kidney cancer [40, 41]. Recently, Shi, Z. G. et al. found that Vimentin was significantly increased in ccRCC and positively associated with tumor stage and pathological grade [42]. Furthermore, Ingels, A et al. demonstrated that Vimentin overexpression is an independent predictor of recurrence, specific and overall survival in nonmetastatic ccRCC [16]. Our results of immunohistochemical staining were similar with these studies. We also validated that Vimentin overexpression was notably correlated with VM in our ccRCC cohort, which was in consistency with what Du, J et al. reported with the pattern of VM and Vimentin expressions in ovarian cancer [18]. These results implied that combining detection of VM and Vimentin would provide reliable basis about biological behavior and prognosis judgments of ccRCC.

## 5. Conclusion

We showed for the first time that cell hypoxia may promote VM formation of RCC cells through upregulation of Vimentin and AXL and downregulation of E-cadherin expressions. Additionally, we proved that targeted Vimentin was sufficient to reduce cell migration and invasion and VM of RCC cells* in vitro*. Not only that, but we also verified the clinical significance and prognostic value of VM and Vimentin expression pattern in ccRCC tissues. However, the underlying mechanism of hypoxia induced activation of Vimentin/AXL axis in RCC VM formation is yet to be determined.

## Figures and Tables

**Table 1:** Relationship between VM and Vimentin expression and clinical parameters in ccRCC.

		VM			Vimentin		
	*n*	Negative	Positive	*p*-value	Low expression (staining index <4)	High expression (staining index ≥4)	*p*-value
*Age (yr)*				0.278			0.052
<55	54	44	10		16	38	
≥55	59	43	16		28	31	
*Gender*				0.731			0.403
Female	36	27	9		12	24	
Male	77	60	17		32	45	
*Position*				0.055			0.060
Left	62	52	10		29	33	
Right	51	35	16		15	36	
*Grade (Differentiation level)*				<0.001			<0.001
Well	88	82	6		44	44	
Moderately and poorly	25	5	20		0	25	
*TNM stage*				<0.001			<0.001
I+II	87	80	7		43	44	
III+IV	26	7	19		1	25	
*Metastasis*				<0.001			<0.001
No	94	87	7		44	50	
Yes	19	0	19		0	19	

## Data Availability

The data used to support the findings of this study are available from the corresponding author upon request.
